# A Case of Unintentional Release of the Watchman FLX Device During Implantation: A Cautionary Tale

**DOI:** 10.7759/cureus.36002

**Published:** 2023-03-10

**Authors:** Ajoe J Kattoor, Christopher Manion, Chee H Kim, Vijay S Iyer

**Affiliations:** 1 Interventional and Structural Cardiology, Kaleida Health/University at Buffalo, Buffalo, USA; 2 Electrophysiology, Kaleida Health/University at Buffalo, Buffalo, USA

**Keywords:** unintentional release of watchman, left atrial appendage closure (laac), atrial fibrillation (af), left atrial appendage occlusion, watchman device, chatgpt

## Abstract

Atrial fibrillation (AF) is a common heart rhythm disorder that increases the risk of stroke and other cardiovascular complications. The Watchman FLX device is a percutaneous device used for the closure of the left atrial appendage in patients with atrial fibrillation and prevents blood clots from forming, thereby decreasing the risk of stroke. However, device dislodgement during the procedure can occur and compromise the effectiveness of the device. In this case report, we present the experience of an 86-year-old man with a history of atrial fibrillation, coronary artery disease, hypertension, and recent gastrointestinal bleeding due to diverticulosis. During the procedure, the device became dislodged prior to the planned release from the shaft due to the difficult anatomy of the appendage, which caused the operator to over-torque and kink the delivery sheath. The kinked sheath prevented the transmission of torque to the appropriate region of the sheath and caused the device to unscrew prematurely. Fortunately, the device was self-deployed in a satisfactory position, and no further intervention was required. The patient did not experience any complications prior to discharge and follow-up echocardiography showed proper positioning of the device. This case highlights the importance of careful technique during the Watchman FLX procedure and the need to replace the delivery sheath if kinking is noted to prevent unintentional dislodgement of the device. In addition, the case was written with the aid of the artificial intelligence-powered language model ChatGPT and demonstrates its benefits as well as calls for caution while using it for medical writing.

## Introduction

Atrial fibrillation (AF) is a common cardiac condition that increases the risk of stroke [1]. Anticoagulation therapy is an essential aspect of AF management to reduce the risk of stroke [2]. However, anticoagulation therapy poses a risk of bleeding. In this context, alternative therapeutic options, such as the Watchman FLX device, have been developed for stroke prevention in patients with AF and for use in patients with a high risk of bleeding. The Watchman FLX device is a left atrial appendage closure device that reduces the risk of stroke by preventing a thrombus from forming in the left atrial appendage [3,4].

We present the case of an 86-year-old man with a medical history of AF and a recent GI bleed due to diverticulosis who underwent Watchman FLX implantation. The procedure was complicated by an unintended release of the device, which occurred due to over-torquing of the delivery sheath. The aim of this case report is to emphasize the importance of avoiding over-torquing of the delivery sheath during Watchman FLX implantation procedures. In addition, the report describes the experience of using the artificial intelligence (AI)-powered language model ChatGPT in medical report writing.

## Case presentation

**Figure 1:**
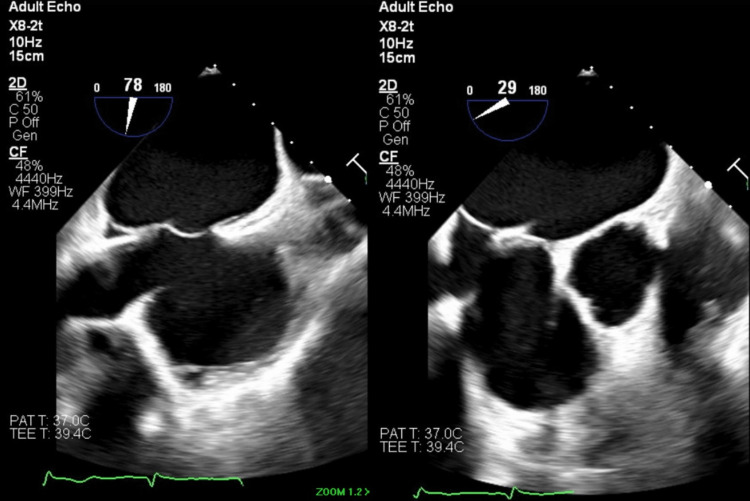
Trans-septal puncture location

**Figure 2:**
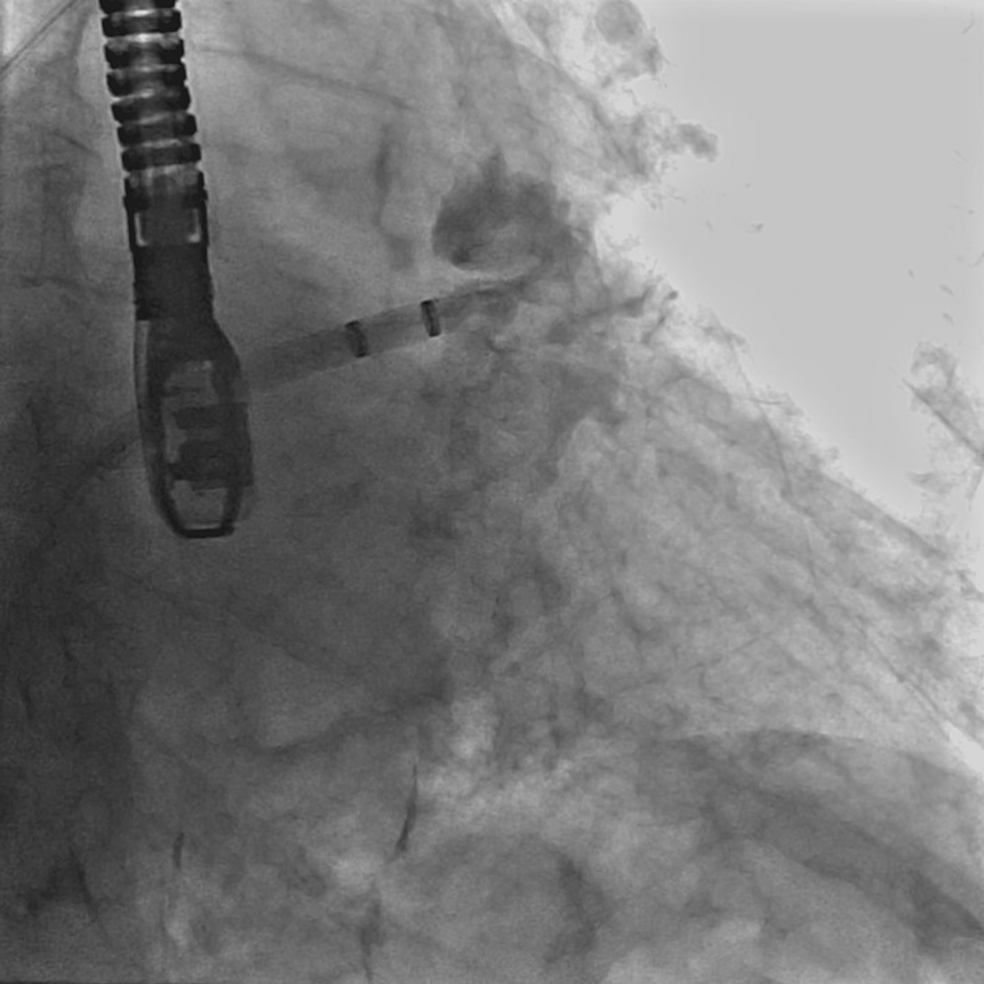
Baseline image of the left atrial appendage Fluoroscopic image (RAO-caudal projection) of the left atrial appendage obtained after contrast injection through the 5-Fr pigtail catheter.

An 86-year-old man with a medical history of atrial fibrillation, coronary artery disease, hypertension, and a recent gastrointestinal bleed due to diverticulosis presented for a Watchman FLX implantation procedure. The physical examination and laboratory data were unremarkable. The procedure was performed through right femoral vein access under transesophageal echocardiogram (TEE) guidance and general anesthesia. A 16-French (Fr) sheath was inserted into the right femoral vein, and an 8-Fr Baylis trans-septal sheath was advanced through the large-bore sheath and into the right atrium over the VersaCross trans-septal guidewire. A trans-septal puncture was performed under TEE guidance using the VersaCross trans-septal guidewire and trans-septal sheath in the standard fashion (Figure 1). The VersaCross trans-septal guidewire was then advanced into the left superior pulmonary vein, and the trans-septal sheath was removed. A 14-Fr Watchman FLX double-curve delivery sheath was advanced into the left atrium over the VersaCross trans-septal guidewire. After the removal of the VersaCross trans-septal guidewire, a 5 Fr pigtail catheter was placed in the left atrial appendage through the Watchman FLX delivery sheath. IV contrast was injected through the pigtail catheter to visualize the left atrial appendage (Figure 2). Echocardiographic and fluoroscopic measurements were collected, and a 27-mm Watchman FLX device was selected.

**Figure 3:**
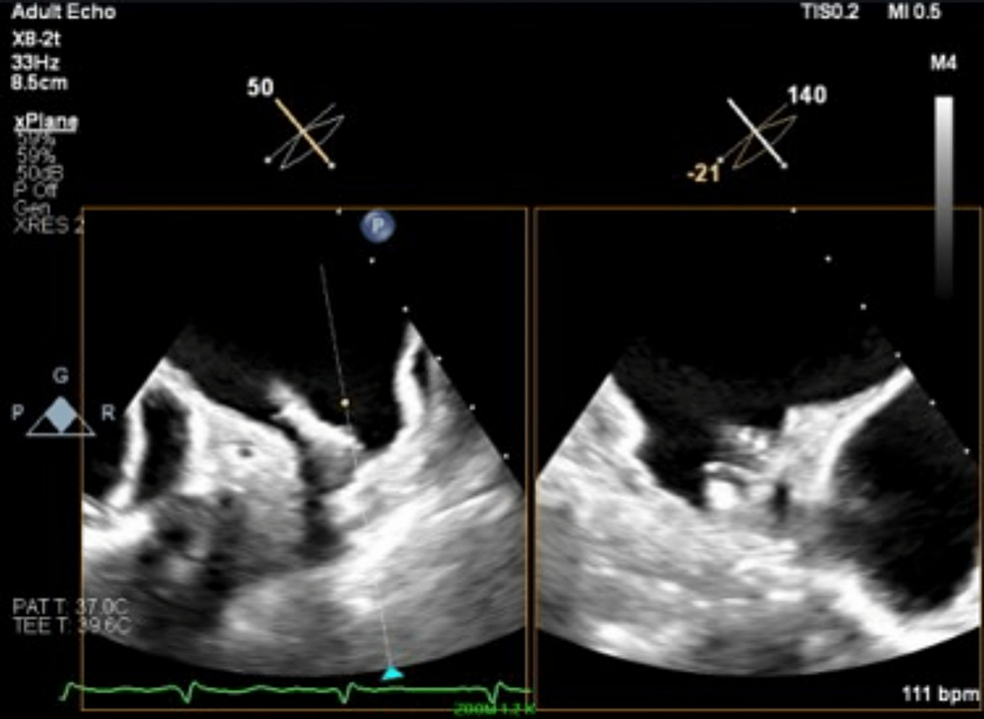
Approach of the Watchman FLX double curve delivery sheath into the left atrial appendage

**Figure 4:**
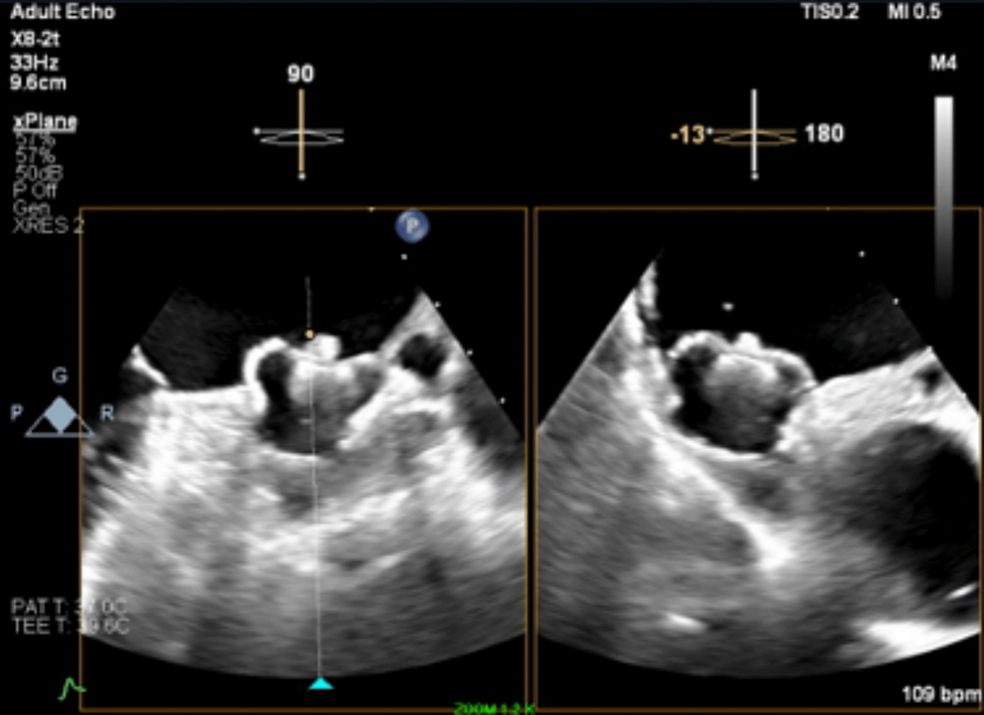
Transesophageal echocardiogram showing final stable position of the Watchman FLX device in the left atrial appendage

The deployment of the Watchman FLX device was challenging due to the patient's anatomy, making it difficult to advance the delivery sheath over the 5-Fr pigtail catheter into the left atrial appendage (Figure 3). Multiple attempts were made with counterclockwise torquing, but the Watchman delivery sheath eventually kinked. At first, it was considered to remove the delivery sheath and use a new device and sheath, but the device was already close to the optimal position in the left atrial appendage by this time, so deployment was attempted. The initial deployment resulted in malposition, so the device was partially recaptured. A total of two complete recaptures and one partial recapture were performed during this process. Further counterclockwise torque was applied to better align the device with the left atrial appendage, and an attempt was made to deploy the device by unsheathing the delivery sheath. But during this time, the device became dislodged from its delivery shaft and was unintentionally implanted (Video 1). The final position was reasonable, and post-delivery compressions were appropriate according to TEE measurements (Figure 4). A tug test for stability could not be performed, but no significant color Doppler flow was observed around the device. A follow-up TEE at 45 days showed no significant complications.

**Video 1:** Unintentional release of the Watchman FLX device from the delivery shaft

## Discussion

Watchman FLX implantation, a procedure for left atrial appendage occlusion, is considered a safe and effective alternative to anticoagulation therapy for stroke prevention in patients with atrial fibrillation [3]. Some of the common complications reported in the literature include procedure-related bleeding, pericardial effusion, device embolization, device leaks, and device thrombosis, which are relatively few in number [4-7]. Cases of device dislodgement reported after left atrial appendage closure are mostly after device implantation. There are no previous reports of unintentional release of the device from its shaft during the delivery of the device due to a kinked delivery sheath.

In our case, the kinked delivery sheath pinned the delivery shaft, and further counterclockwise torquing provided to the Watchman FLX sheath while in a partially recaptured device position caused the device to unscrew from the delivery shaft. The device was attempted to be deployed by unsheathing the delivery sheath without realizing that the shaft had already unscrewed and hence been dislodged/released unintentionally. Fortunately, the device landed in a satisfactory position in the left atrial appendage, and the patient did not have any complications.

Dimitri et al. had previously reported a case of inability to retract the watchman device from the left atrium due to it getting stuck in the kinked delivery sheath, which was resolved by straightening the kink with an Amplatz Super Stiff wire [8]. This case highlights the importance of careful technique during the Watchman FLX procedure and the need to replace the delivery sheath if kinking is noted to prevent unintentional unscrewing and dislodgement/release of the device. This is the first case encountered at our institution where unusual torque on the sheath affected the screw mechanism holding the device onto the shaft.

AI-powered language model ChatGPT was used to aid in the writing of this manuscript [9]. First, the model was given the core content and asked to write a report and suggest a title (Appendix Figure 5). The system appeared to comprehend the information provided, even though there are no previous reports of such a case, and suggested an appropriate title, which was used in the article. It was helpful in writing the introduction, abstract, and conclusion sections (Appendix Figure 6). However, the model was unable to write a meaningful discussion section. The use of ChatGPT reduced the time needed to write the manuscript and appeared to be very useful for researchers who are not proficient in the English language. However, the alarming part is that the citations provided by ChatGPT were not accurate, and there was no real article with that information when verified using PubMed and Google searches (Appendix Figure 7). Therefore, it is difficult to determine the authenticity of the content provided by ChatGPT, especially for laypeople who are not proficient in a subject. The use of ChatGPT without oversight in writing academic literature can lead to the accumulation of misinformation and the creation of fraudulent articles. The frightening aspect is that it will be difficult to differentiate between real and AI-generated articles once the internet is flooded with such content. Hence, it is important to devise a tool that can identify articles written with ChatGPT. It is also important to create a tool that can verify medical information that goes into the AI model so that the model learns using accurate information. In short, ChatGPT is useful as a language model, but at its current level, it should be limited to use in English language editing in the medical literature.

## Conclusions

In conclusion, this case report highlights the challenges and complexities associated with the Watchman FLX procedure. The cause of the unintentional release of the device in this patient was due to difficulty in placement and over-torquing of the delivery sheath, which resulted in kinking and unscrewing of the device from the shaft. Despite the adverse event, the patient did not experience any significant complications, and the device was properly positioned at follow-up echocardiography. This case underscores the importance of careful technique and attention to detail during the Watchman FLX procedure to prevent device dislodgement/unintentional release and ensure optimal patient outcomes. Additionally, the report highlights both the benefits and ‘cautionary tale’ of using AI-powered models like ChatGPT for medical writing.

## Figures and Tables

**Figure 5:**
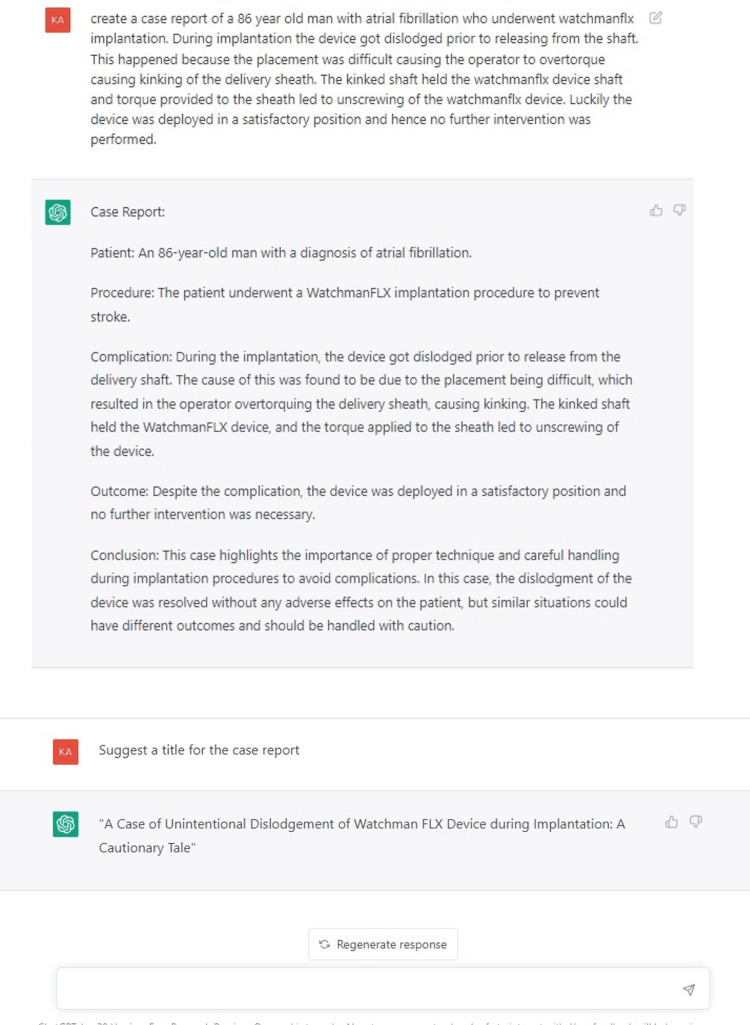
Initial instructions to ChatGPT ChatGPT was able to comprehend and provide a small case report and suggest a title for the article.

**Figure 6:**
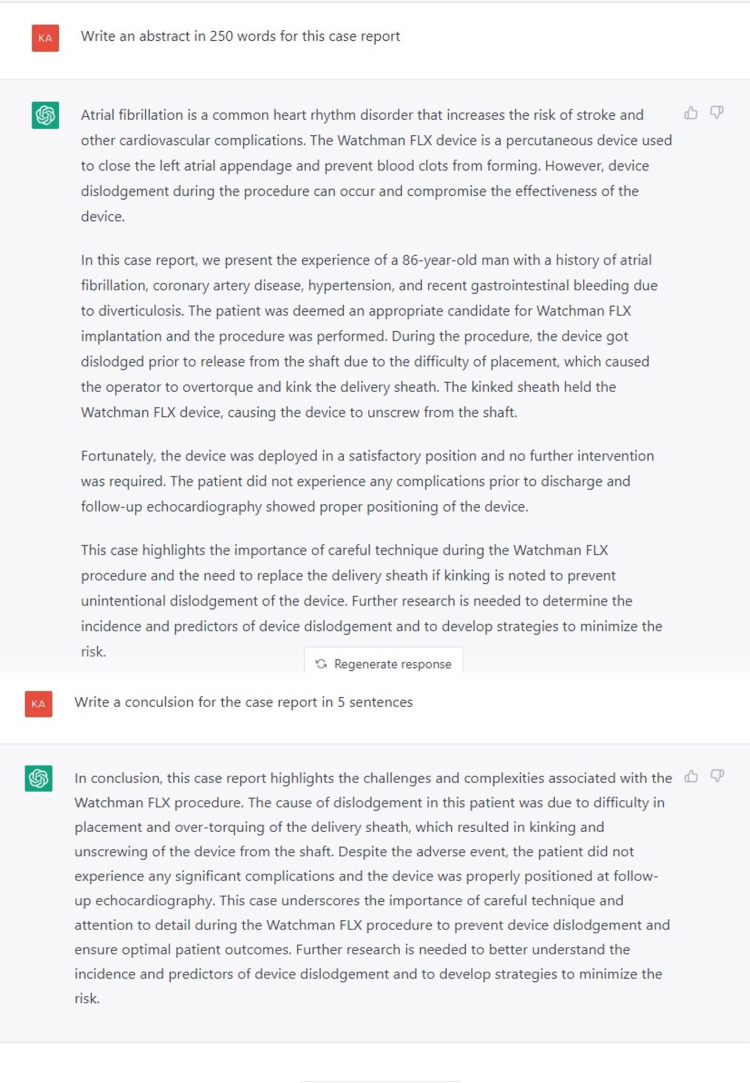
Generating abstract and conclusion ChatGPT was able to use its skills to generate an abstract and conclusion section for the article with reasonable accuracy.

**Figure 7:**
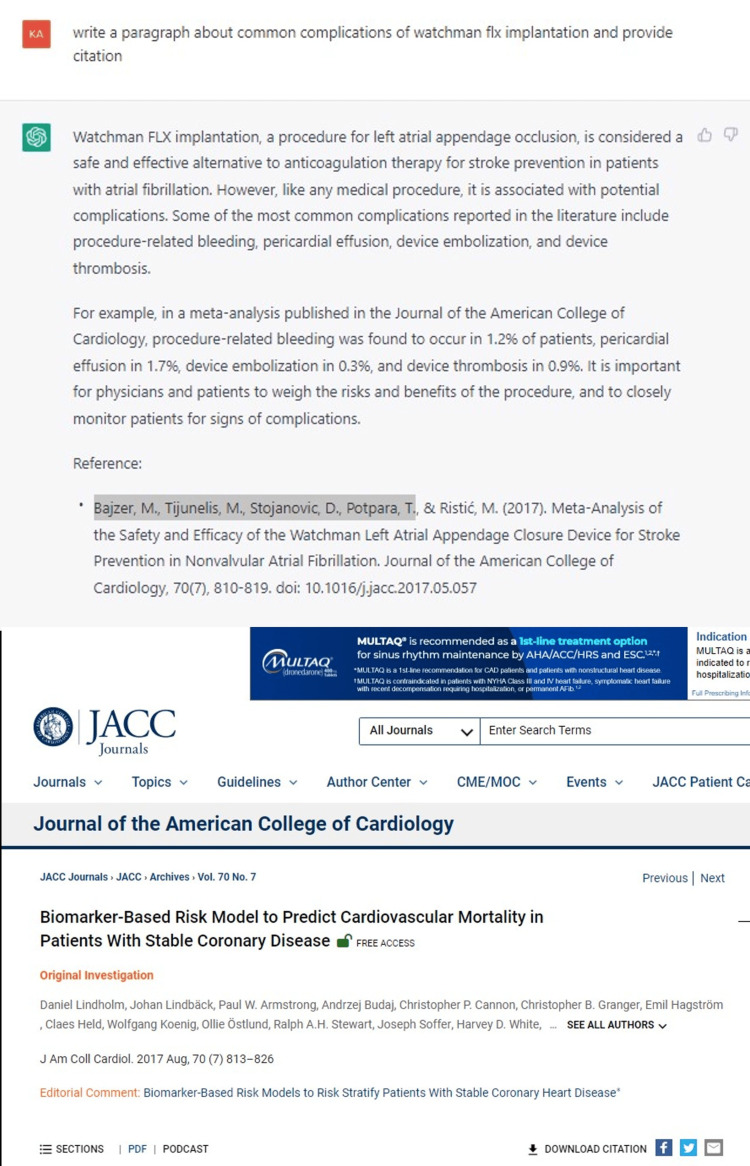
Highly inaccurate citation information by ChatGPT Example of ChatGPT providing inaccurate citation. The lower portion of the figure shows the actual article obtained following the citation provided by ChatGPT. Additionally, the article cited by ChatGPT could not be found after google and PubMed search using the title and authors.
